# Supplementary material for: T cell receptor beta germline variability is revealed by inference from repertoire data
Source: Genome Med. 2022 Jan 7;14:2. doi: 10.1186/s13073-021-01008-4 (PMC8740489; doi:10.1186/s13073-021-01008-4)
Supplement: Supplementary file 1 — Additional file 1 Supplementary materials: TRB germline variability is revealed by inference from repertoire data Supplementary figures 1–14: Figure S1 Unique sequence distribution in the four data sets. Figure S2 TRBD2 and TRBJ1-6 genotypes and gene usage frequencies. Figure S3 5’ UTR nucleotide sequences of TRBV genes. Figure S4 5’ UTR nucleotide sequences of TRBV genes. Figure S5 DS2 TRB genotype heatmap. Figure S6 TRBV6-2/TRBV6-3 usage correlates to the existence of TRBV4-3and TRBV3-2 in DS1. Figure S7 Linkage disequilibrium in TRBD2 haplotype. Figure S8 Genomic correlation between TRBD2 and TRBJ1-6 in DS3. Figure S9 TRBD and TRBJ usage out of the non-functional sequences corresponds to TRBD2 genotype in DS4. Figure S10 Heatmap of TRBV4-3 genotype correlates to the TRBV7-2 genotype in DS3. Figure S11 TRBV gene usage according to the TRBV7-2 genotype in DS3. Figure S12 TRBV haplotypes for 10 individuals from DS1. Figure S13 TRBV SNPs distribution. Figure S14 IGHV SNPs distribution. Supplementary tables 1–11: Table S1 DS2 data set sources citation. Table S2 BIOMED-2 allele patterns. Table S3 Adaptive allele patterns. Table S4 The distribution parameters of the TRBD2*01 fraction accordingto the TRBD2 genotype group in DS4. Table S5 Previously unknown alleles comparison. Table S6 Incomplete allele extensions table. Table S7 5’ UTR variants. Table S8 5’ UTR undocumented sequences. Table S9 Undocumented allele verification in artificial partial libraries. Table S10 Previously unknown alleles comparison. Table S11 Previously unknown alleles comparison. [file 13073_2021_1008_MOESM1_ESM.pdf]

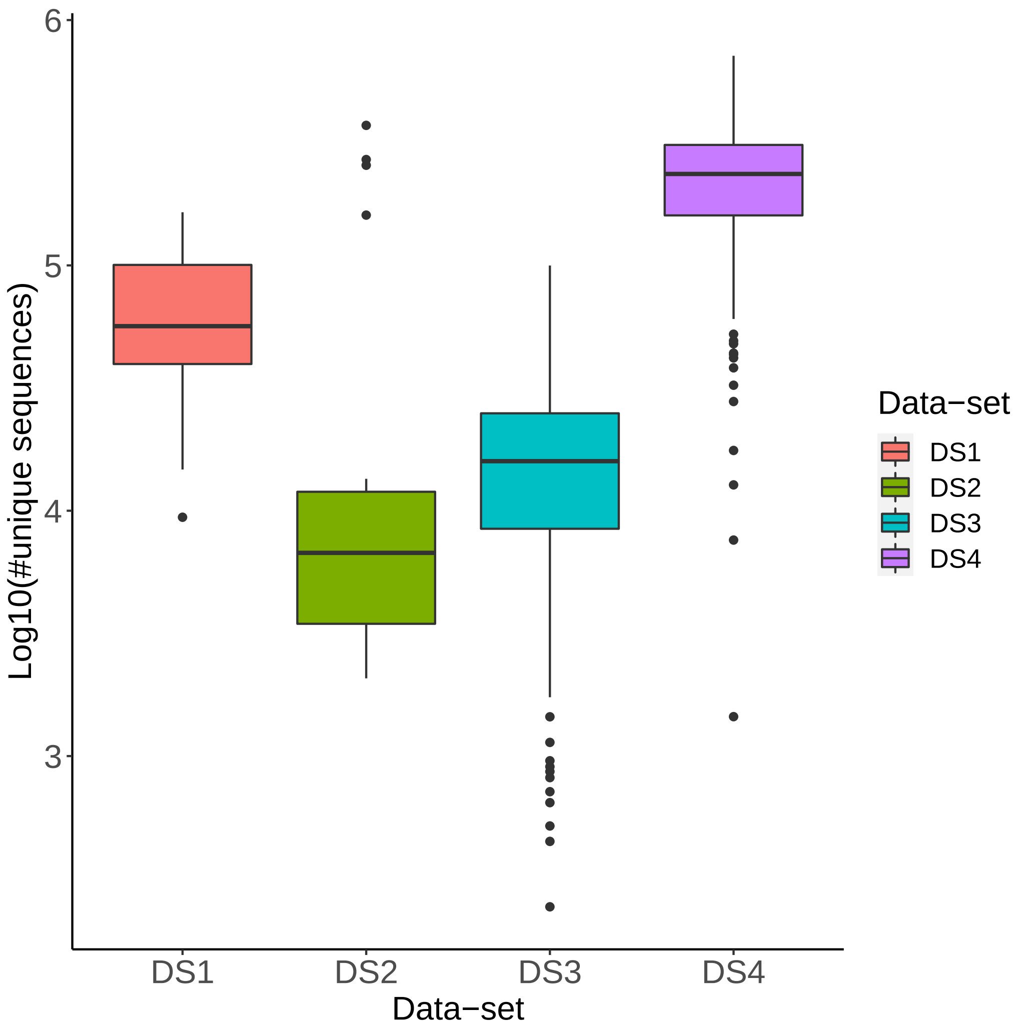

Fig S1: **Unique sequence distribution in the four data-sets** The X-axis represents the four data-sets. The Y-axis is the number of unique sequences by log10.

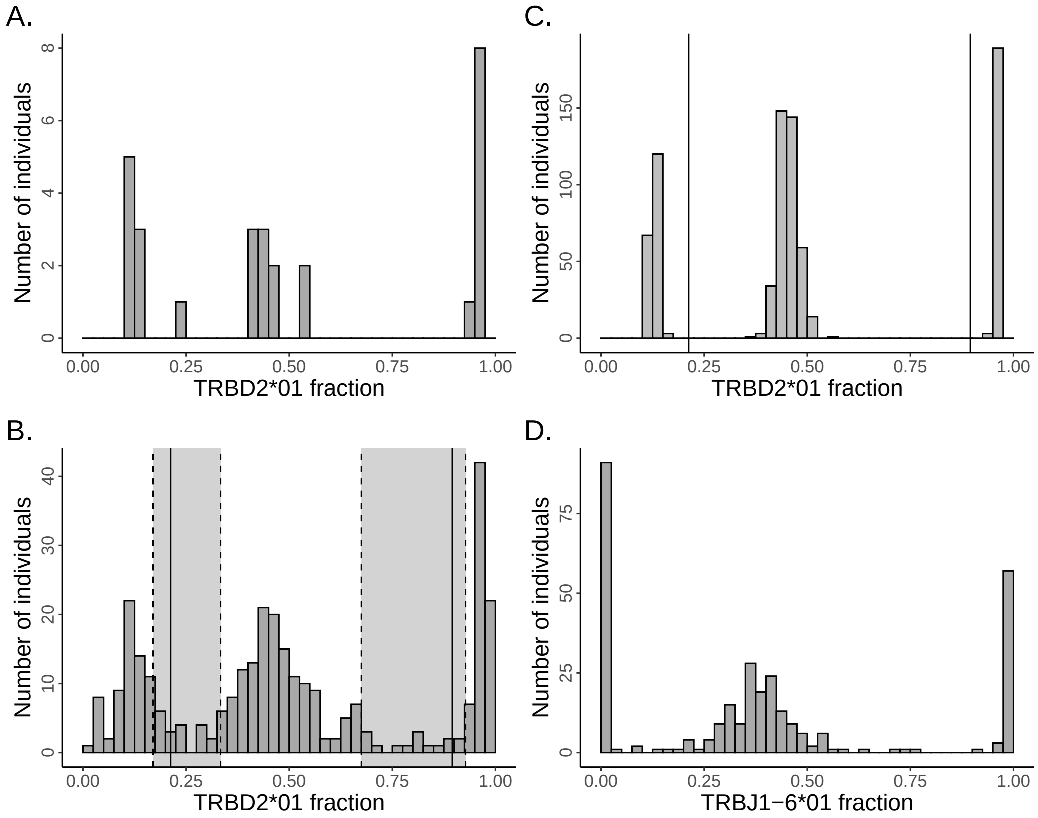

Fig S2: **TRBD2 and TRBJ1-6 genotypes and gene usage frequencies.** (A) The frequency of TRBD2*01 usage, as a fraction of total D2 usage in 28 individuals from DS1. Homozygous and heterozygous genotypes can be inferred from the distribution. (B) The frequency of TRBD2*01 usage, as a fraction of total D2 usage in 313 individuals from DS3. Solid vertical lines indicate boundaries between homozygous and heterozygous individuals, as calculated from analysis of DS4. The genotypes of individuals whose frequencies fall within the shaded regions cannot be inferred with confidence. (C) The frequency of TRBD2*01 usage, as a fraction of Total TRBD2 usage in 786 individuals from DS4. Solid vertical lines indicate boundaries between homozygous and heterozygous individuals. (D) The frequency of TRBJ1-6*01 usage, as a fraction of Total TRBJ1-6 usage in 313 individuals from DS3.

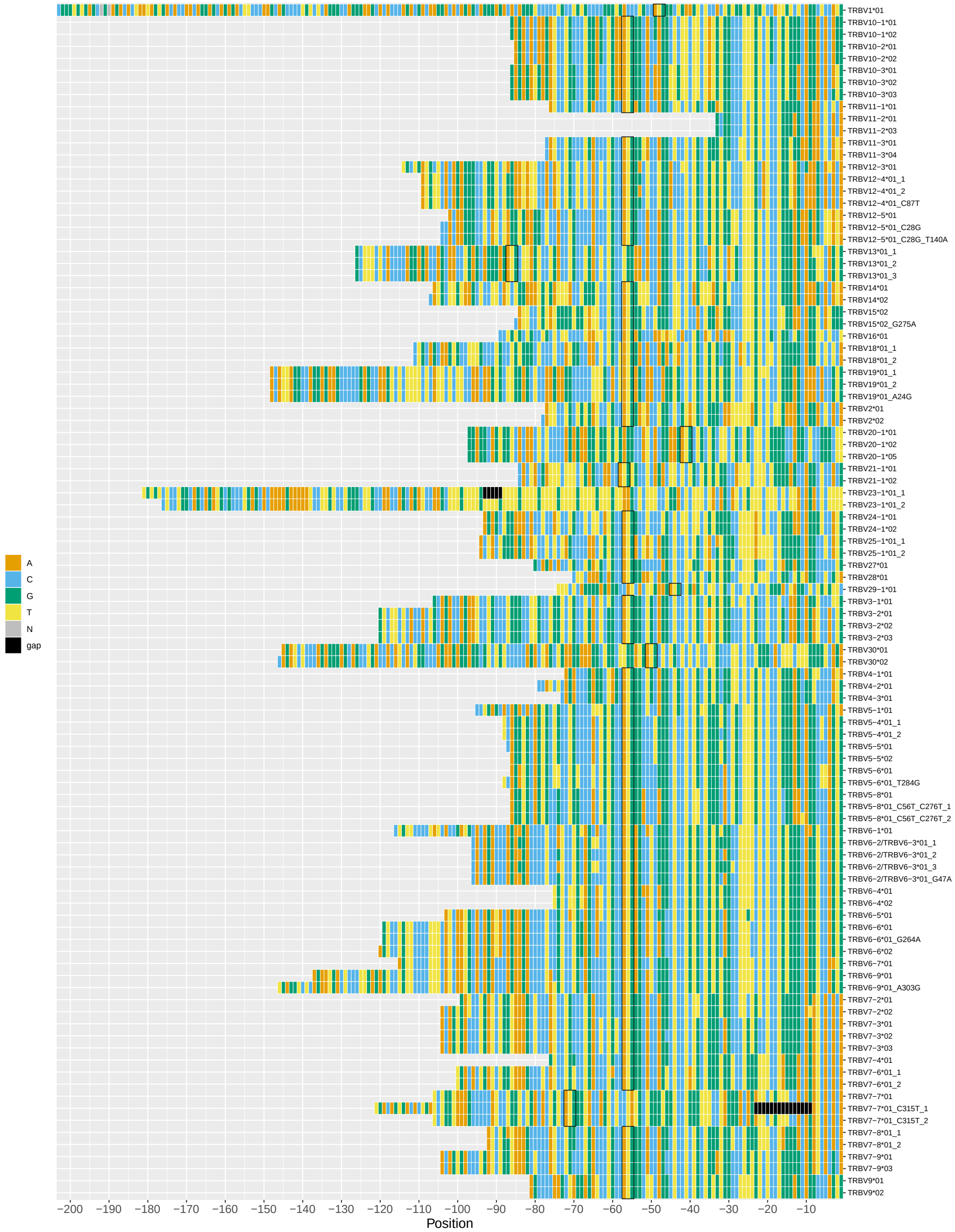

Fig S3: **5′ UTR nucleotide sequences of TRBV genes.** Each row is a consensus sequence for an allele. The column positions are numbered from the start of the FR1 region. Start codons are indicated with black frames.

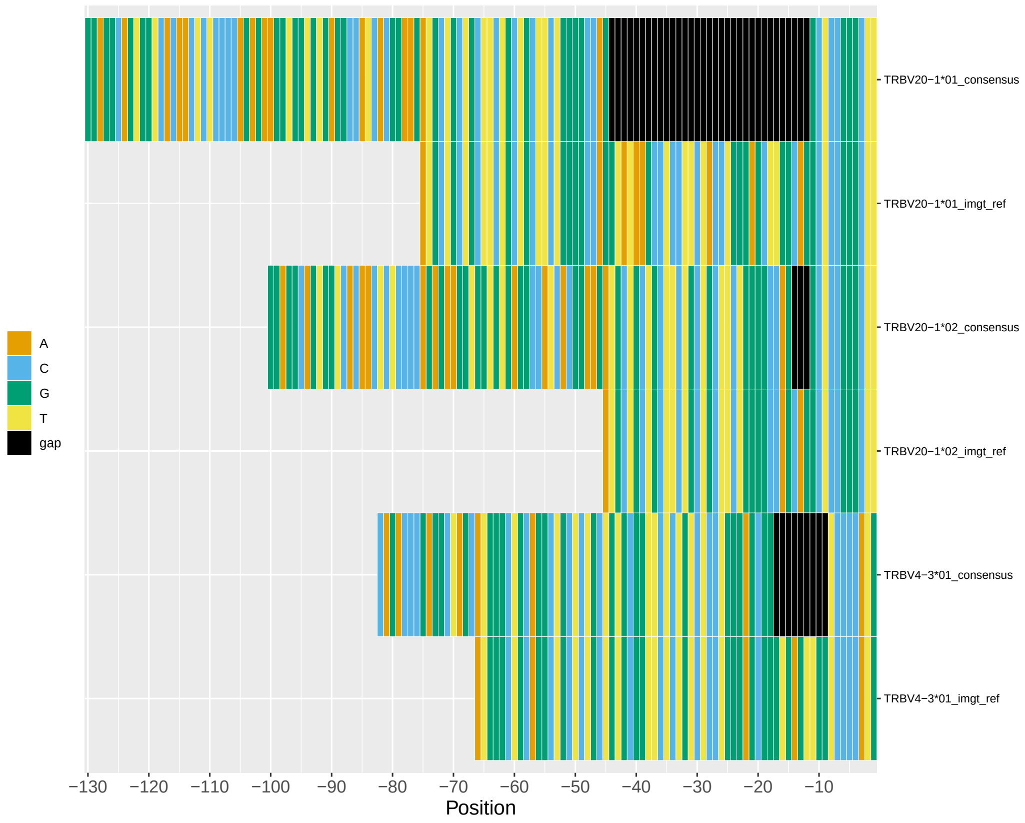

Fig S4: **5′ UTR nucleotide sequences of TRBV genes.** Each row is or a consensus sequence for an allele or the IMGT reference sequence for the allele. The column positions are numbered from the start of the FR1 region. The gaps for the consensus sequences were opened between the end of the L-PART1 to the beginning of the L-PART2.

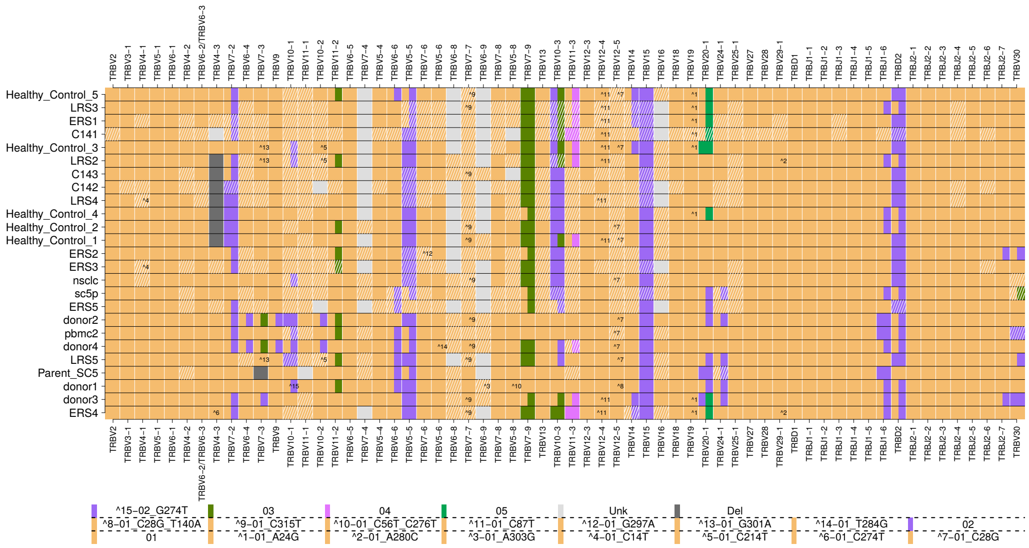

Fig S5: **DS2 TRB genotype heatmap.** Each row is an individual, each column is a gene sorted by locus. Colors correspond to alleles.

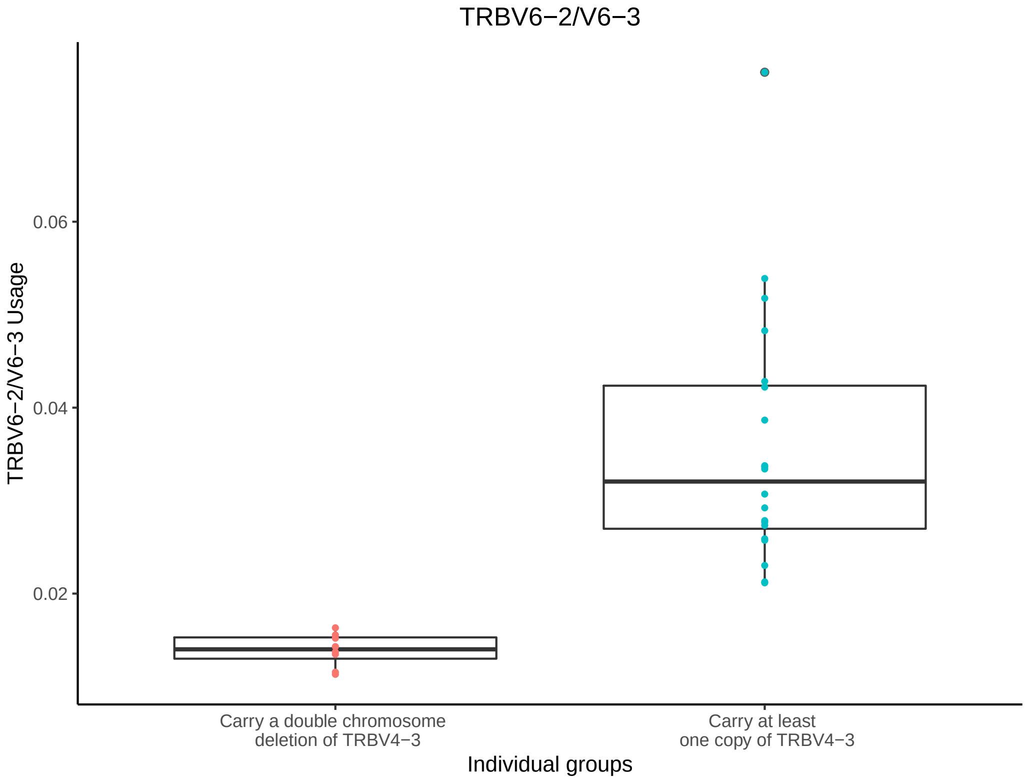

Fig S6: **TRBV6-2/TRBV6-3 usage correlates to the existence of TRBV4-3 and TRBV3-2 in DS1.** The X-axis represents the two groups of individuals, the Y-axis is the TRBV6-2/TRBV6-3 usage out of all the TRBV genes, and the colors correspond to the individual group.

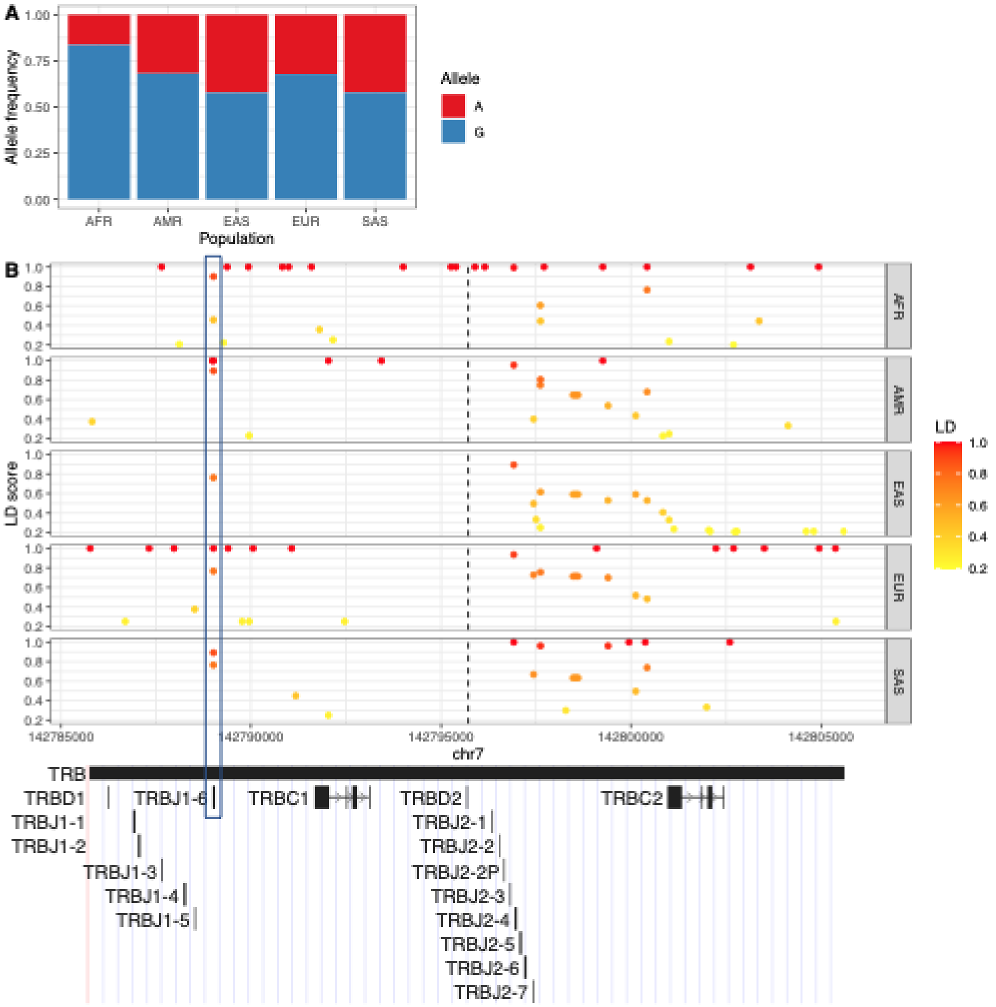

Fig S7: **Linkage disequilibrium in TRBD2 haplotype.** Whole genome sequencing was used to haplotype, based on TRBD2 alleles as anchors, and pinpoint potential linkage disequilibrium SNPs within the TRBD-TRBC2 chr7 region. (A) The allelic frequency of TRBD2 within each population. The Y-axis is the allele frequency, the x-axis is the different population groups. The colors represent the allele, blue for G and red for A. (B) Genome data viewer for the TRBD-TRBC2 chr7 region. The upper panel shows the LD score of each variant per position and for each population group. Each row is a population group, and each column is a genomic position in chr7. Y-axis is the LD score, and each dot is a genomic variant. The color scale represents the LD score value. The lower panel shows the genomic annotations for the selected chr7 region. The annotations' locations correspond to the upper panel x-axis genomic positions. The dashed line points to the SNP position that differentiates between the TRBD2 alleles, and the rectangle marks TRBJ1-6.

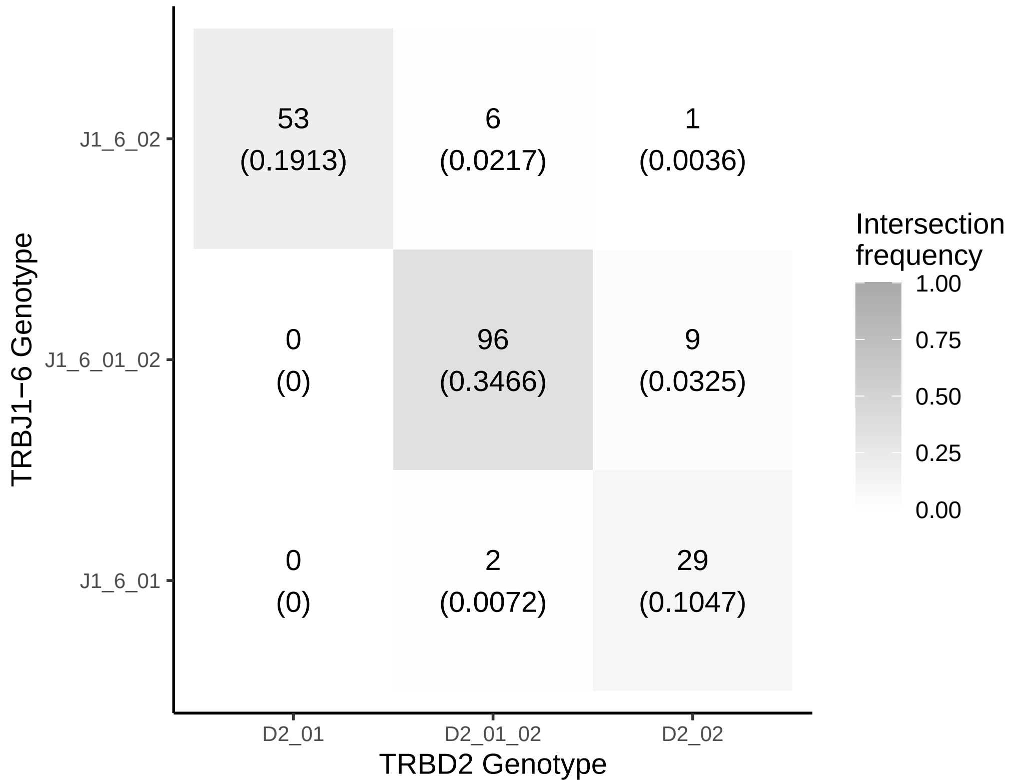

Fig S8: **Genomic correlation between TRBD2 and TRBJ1-6 in DS3.** The number and proportions of individuals in DS3 as observed with different TRBJ1-6 and TRBD2 genotypes.

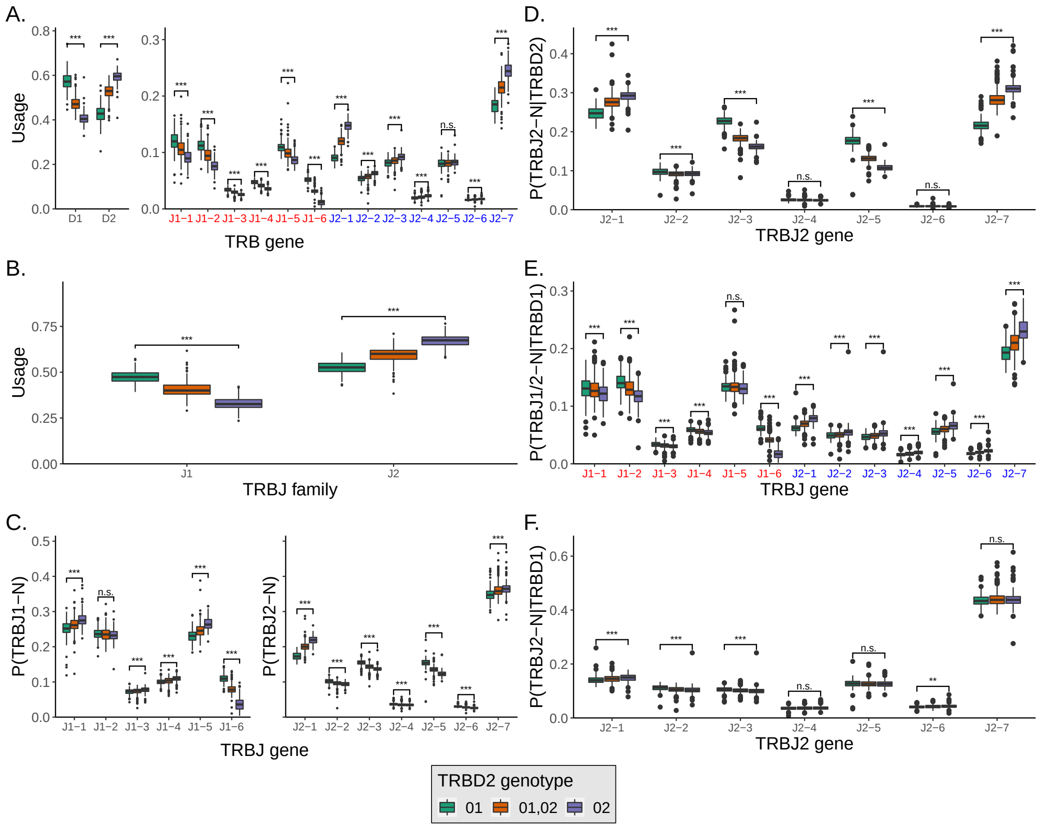

Fig S9: **TRBD and TRBJ usage out of the non-functional sequences corresponds to TRBD2 genotype in DS4.** (A) TRBD and TRBJ gene usage in DS4 individuals with different TRBD2 genotype. TRBJ genes are shown along the X-axis in the order in which they are found in the genome. (B) The TRBJ family usage in DS4 individuals with different TRBD2 genotypes. TRBJ families are shown along the X-axis in the order in which they appear in the genome. (C) TRBJ gene usage normalized according to the TRBJ "family" usage in DS4 individuals with different TRBD2 genotype. TRBJ genes are shown along the X-axis in the order in which the genes are found in the genome. (D) The fraction of TRBJ2 genes out of the sequences that were assigned to TRBD2 and were longer than 7nt. (E) The fraction of TRBJ2 genes out of the sequences that were assigned to TRBD1 and were longer than 7nt. TRBJ genes are shown along the X-axis in the order in which they are found in the genome. (F) The fraction of TRBJ genes out of the sequences that were assigned to TRBD1 and were longer than 7nt. TRBJ genes are shown along the X-axis in the order in which they are found in the genome. The boxes' colors correspond to the TRBD2 genotype. Statistical significance was determined using a Mann-Whitney test and adjusted by Bonferroni correction (n.s. - not significant, * - p < 0.05, ** - p < 0.01, and *** - p < 0.001).

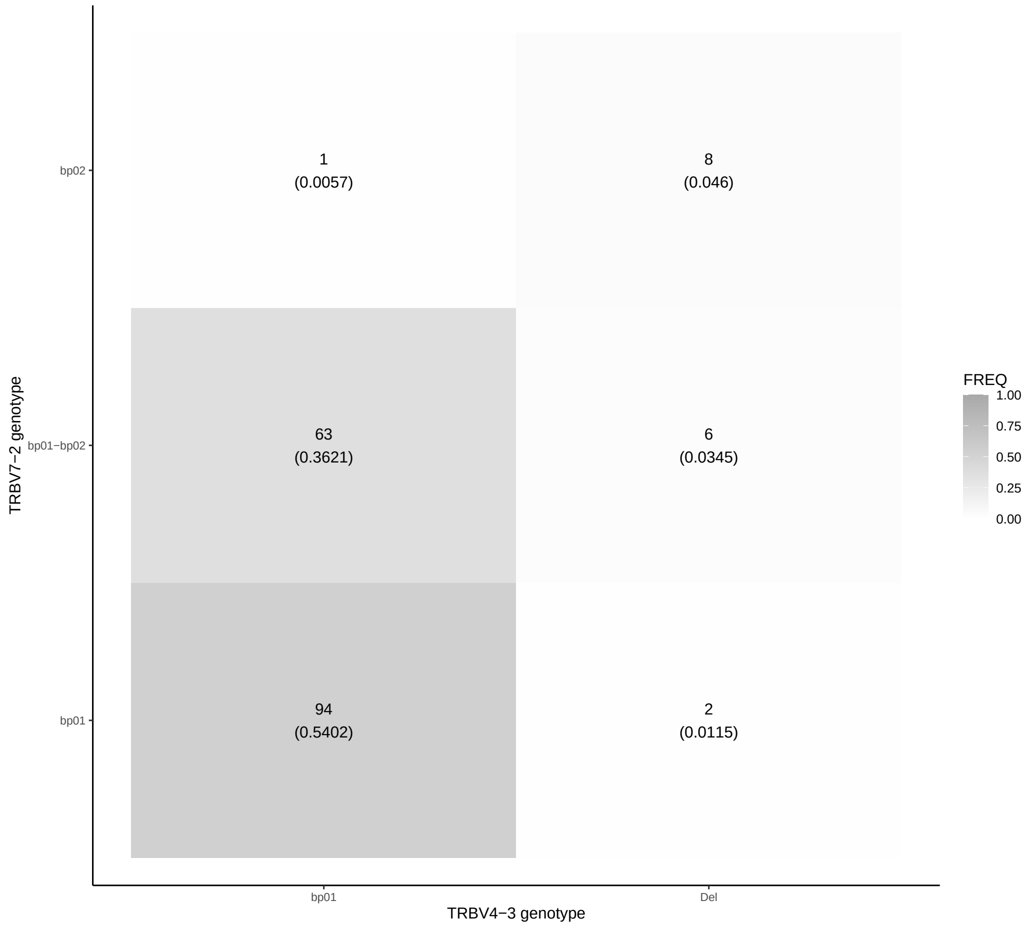

Fig S10: **Heatmap of TRBV4-3 genotype correlates to the TRBV7-2 genotype in DS3.** The X-axis represents the TRBV7-2 genotypes, the Y-axis is the TRBV4-3 genotypes, the values in each block are the number of individuals with the same inferred genotypes, and in brackets is the frequency of individuals who appear in this comparison.

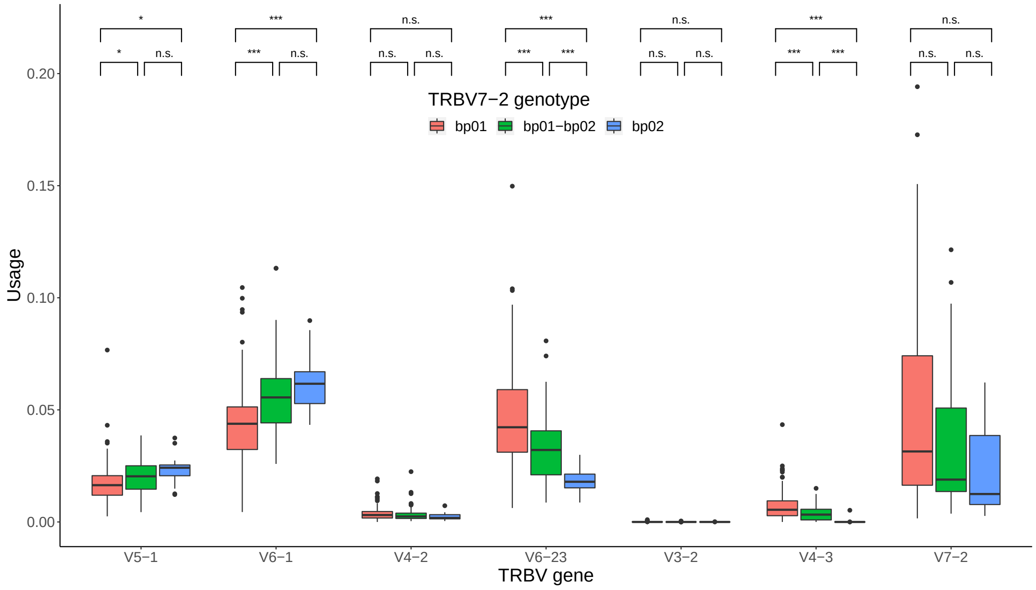

Fig S11: **TRBV gene usage according to the TRBV7-2 genotype in DS3.** Colors correspond to the TRBV7-2 genotype group. Statistical significance was determined using a Mann-Whitney test and adjusted by Bonferroni correction (see Methods; n.s. - not significant, * - $p < 0.05$, ** - $p < 0.01$, and *** - $p < 0.001$).

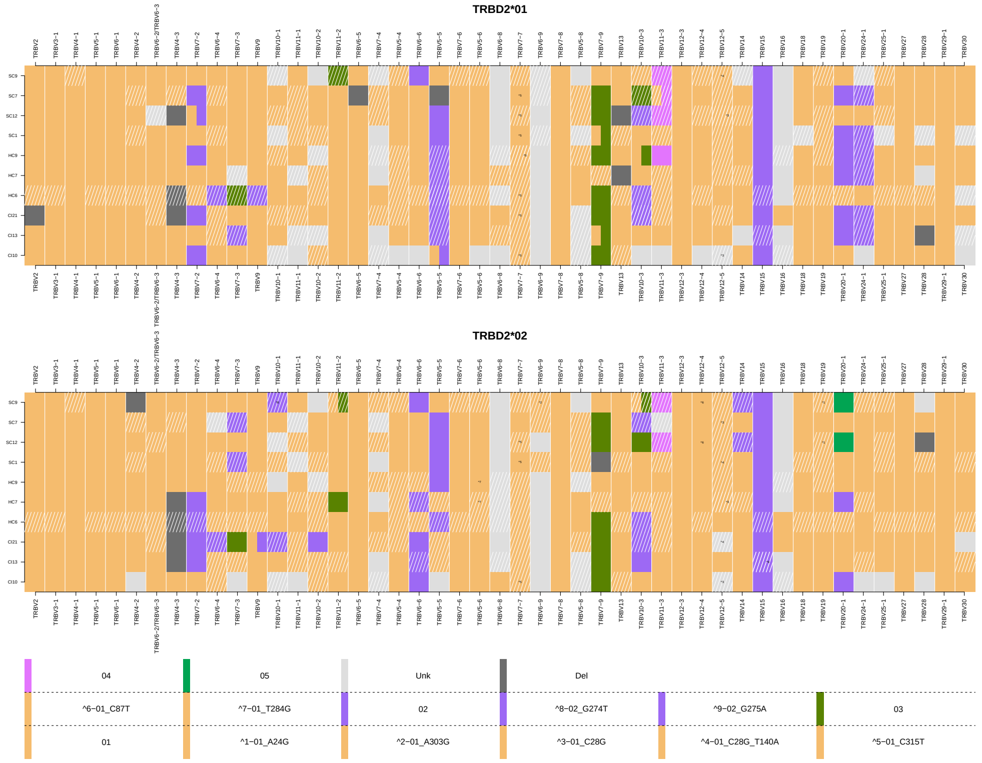

Fig S12: **TRBV haplotypes for 10 individuals from DS1.** The upper and lower panels show the TRBV haplotypes anchored with TRBD2*01 and TRBD2*02, respectively. Each row is an individual's haplotype, and each column is a V gene call. The colors correspond to the V alleles and the tile annotations correspond to the undocumented allele variations.

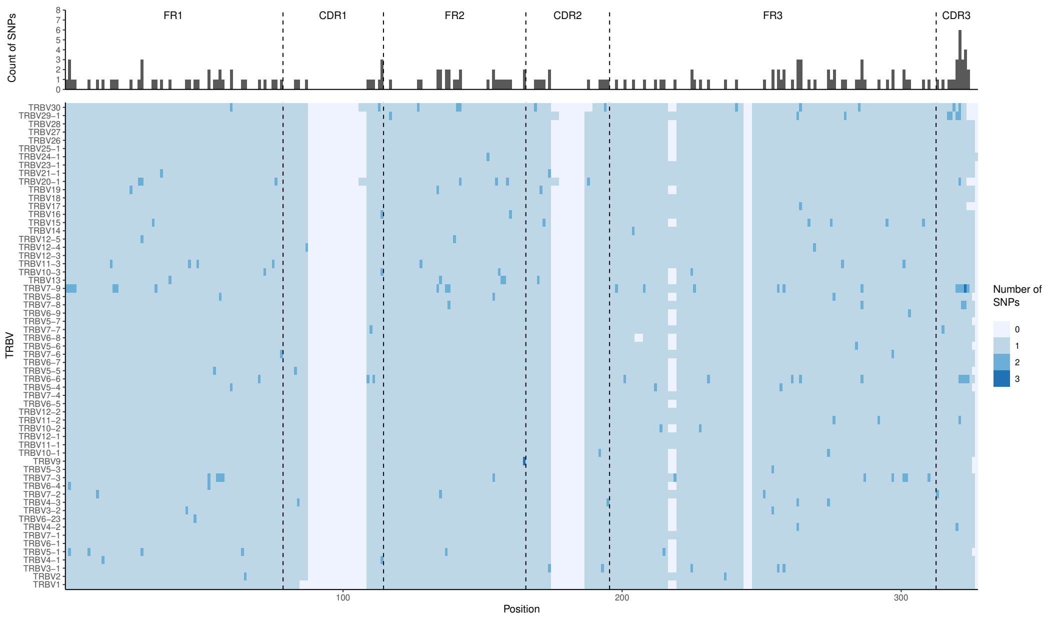

Fig S13: **TRBV SNPs distribution.** The top panel show the SNPs distribution across the V regions (FRX, CDRX) and position. The Y axis is the sum of the SNPs per the position in the X axis. The bottom panel show the distribution of the SNPs per V gene. The Y axis is the different V genes, the X axis is the positions across the V. The color scale represents the number of SNPs observed .

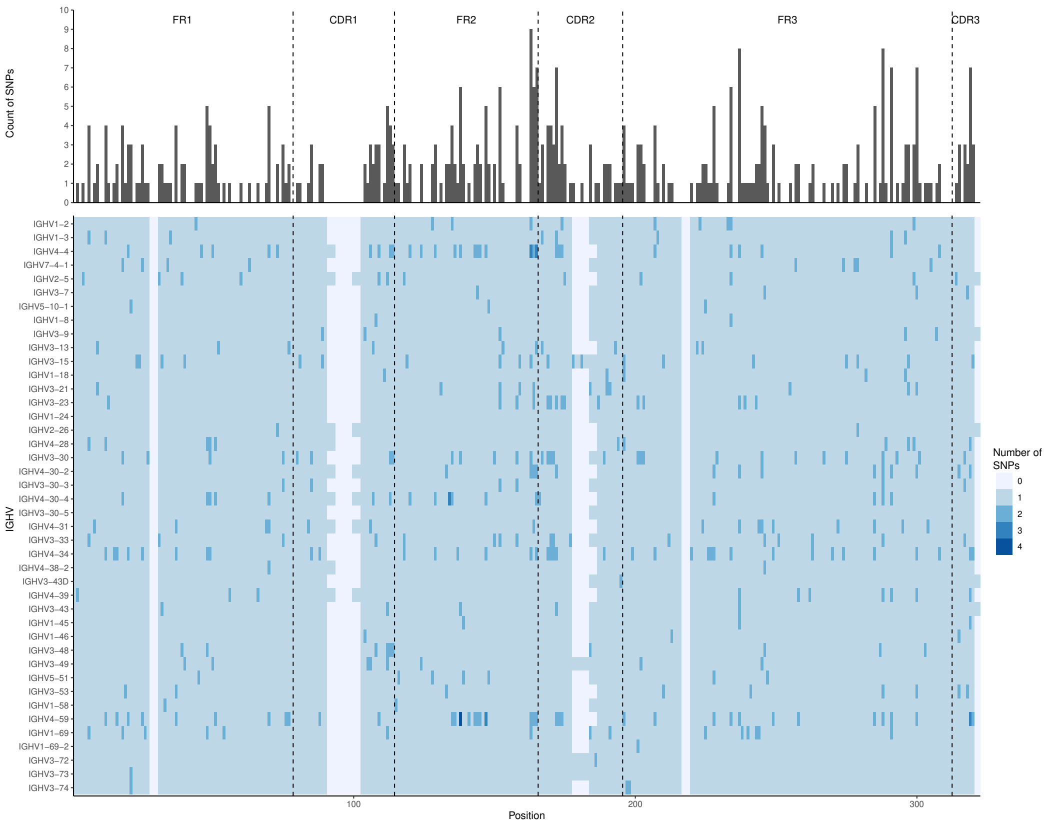

Fig S14: **IGHV SNPs distribution.** The top panel show the SNPs distribution across the V regions (FRX, CDRX) and position. The Y axis is the sum of the SNPs per the position in the X axis. The bottom panel show the distribution of the SNPs per V gene. The Y axis is the different V genes, the X axis is the positions across the V. The color scale represents the number of SNPs observed .

Table S1: **DS2 sources and citations.** The "Individuals" column indicates the names given to the individuals within the source. The "Citations" column indicates the citation of the data-sets that were collected.

Source	Individuals	Citations
10x Genomics [1]	sc5p	Human T cells from a Healthy Donor 1k cells (v2), Single Cell Immune Profiling Dataset by Cell Ranger 5.0.0, 10x Genomics, (2020, November 19).
	Parent_SC5	PBMCs from a Healthy Donor: Paired T Cell Receptor, Single Cell Immune Profiling Dataset by Cell Ranger 4.0.0, 10x Genomics, (2020, July 7).
	donor1, donor2, donor3, and donor4	CD8+ T cells of Healthy Donor 1-4, Single Cell Immune Profiling Dataset by Cell Ranger 3.0.2, 10x Genomics, (2019, May 9).
	pbmc2	PBMCs of a healthy donor - TCR enrichment from amplified cDNA, Single Cell Immune Profiling Dataset by Cell Ranger 3.0.0, 10x Genomics, (2018, November 19).
	nsclc	NSCLC tumor - TCR enrichment from amplified cDNA, Single Cell Immune Profiling Dataset by Cell Ranger 3.0.0, 10x Genomics, (2018, August 1).
GEO [3]	C141, C142, and C143	Liao et al. [8]
EGA [6]	ERS1, ERS2, ERS3, ERS4, ERS5, Healthy_Control_1, Healthy_Control_2, Healthy_Control_3, Healthy_Control_4, Healthy_Control_5, LRS2, LRS3, LRS4, and LRS5	Wen et al. [11]

Table S2: **BIOMED-2 allele patterns.**

GENE	PATTERN	ALLELES
TRBV1	bp01	01
TRBV10-1	bp01	01
TRBV10-1	bp02	02
TRBV10-2	bp01	01
TRBV10-2	bp02	02
TRBV10-3	bp01	01, 02, 04
TRBV10-3	bp02	03
TRBV11-1	bp01	01
TRBV11-2	bp01	01
TRBV11-2	bp02	02
TRBV11-2	bp03	03
TRBV11-3	bp01	01, 02, 04
TRBV11-3	bp02	03
TRBV12-3/TRBV12-4	bp01	TRBV12-4*01, TRBV12-3*01
TRBV12-3/TRBV12-4	bp02	TRBV12-4*02
TRBV12-5	bp01	01
TRBV13	bp01	01
TRBV13	bp02	02
TRBV14	bp01	01
TRBV14	bp02	02
TRBV15	bp01	01
TRBV15	bp02	02
TRBV15	bp03	03
TRBV16	bp01	01, 02
TRBV16	bp02	03
TRBV18	bp01	01
TRBV19	bp01	01
TRBV19	bp02	02, 03
TRBV2	bp01	01, 02
TRBV2	bp02	03
TRBV20-1	bp01	01, 02
TRBV20-1	bp02	03
TRBV20-1	bp03	04, 06
TRBV20-1	bp04	05

TRBV20-1	bp05	07
TRBV20/OR9-2	bp01	01
TRBV20/OR9-2	bp02	02
TRBV20/OR9-2	bp03	03
TRBV21-1	bp01	01
TRBV21-1	bp02	02
TRBV21/OR9-2	bp01	01
TRBV23-1	bp01	01
TRBV23/OR9-2	bp01	01
TRBV23/OR9-2	bp02	02
TRBV24-1	bp01	01
TRBV24-1	bp02	02
TRBV24/OR9-2	bp01	01
TRBV25-1	bp01	01
TRBV26	bp01	01
TRBV26/OR9-2	bp01	01, 02
TRBV27	bp01	01
TRBV28	bp01	01
TRBV29-1	bp01	01
TRBV29-1	bp02	02
TRBV29-1	bp03	03
TRBV29/OR9-2	bp01	01
TRBV29/OR9-2	bp02	02
TRBV3-1	bp01	01
TRBV3-1	bp02	02
TRBV3-2	bp01	01
TRBV3-2	bp02	02, 03
TRBV30	bp01	01
TRBV30	bp02	02
TRBV30	bp03	03
TRBV30	bp04	04
TRBV30	bp05	05
TRBV4-1	bp01	01, 02
TRBV4-2	bp01	01
TRBV4-2	bp02	02
TRBV4-3	bp01	01, 04

TRBV4-3	bp02	02
TRBV4-3	bp03	03
TRBV5-1	bp01	01
TRBV5-1	bp02	02
TRBV5-3	bp01	01
TRBV5-3	bp02	02
TRBV5-4	bp01	01, 03
TRBV5-4	bp02	02
TRBV5-4	bp03	04
TRBV5-5	bp01	01, 02, 03
TRBV5-6	bp01	01
TRBV5-7	bp01	01
TRBV5-8	bp01	01
TRBV5-8	bp02	02
TRBV6-1	bp01	01
TRBV6-2/TRBV6-3	bp01	TRBV6-2*01, TRBV6-3*01
TRBV6-4	bp01	01, 02
TRBV6-5	bp01	01
TRBV6-6	bp01	01, 03
TRBV6-6	bp02	02
TRBV6-6	bp03	04
TRBV6-6	bp04	05
TRBV6-7	bp01	01
TRBV6-8	bp01	01
TRBV6-9	bp01	01
TRBV7-1	bp01	01
TRBV7-2	bp01	01, 04
TRBV7-2	bp02	02
TRBV7-2	bp03	03
TRBV7-3	bp01	01
TRBV7-3	bp02	02
TRBV7-3	bp03	03
TRBV7-3	bp04	04
TRBV7-3	bp05	05
TRBV7-4	bp01	01
TRBV7-6	bp01	01, 02

TRBV7-7	bp01	01, 02
TRBV7-8	bp01	01
TRBV7-8	bp02	02
TRBV7-8	bp03	03
TRBV7-9	bp01	01, 02, 03
TRBV7-9	bp02	04
TRBV7-9	bp03	05
TRBV7-9	bp04	06
TRBV7-9	bp05	07
TRBV9	bp01	01
TRBV9	bp02	02
TRBV9	bp03	03

Table S3: **Adaptive allele patterns.**

GENE	PATTERN	ALLELES
TRBV10-1	ap01	01, 02
TRBV10-2	ap01	01, 02
TRBV10-3	ap01	01, 02, 03, 04
TRBV11-1	ap01	01
TRBV11-2	ap01	01, 03
TRBV11-2	ap02	02
TRBV11-3	ap01	01, 02, 04
TRBV11-3	ap02	03
TRBV12-1	ap01	01
TRBV12-2	ap01	01
TRBV12-3/TRBV12-4	ap01	TRBV12-4*01, TRBV12-4*02, TRBV12-3*01
TRBV12-5	ap01	01
TRBV13	ap01	01, 02
TRBV14	ap01	01, 02
TRBV15	ap01	01
TRBV15	ap02	02
TRBV15	ap03	03
TRBV16	ap01	01, 02, 03
TRBV17	ap01	01, 02
TRBV18	ap01	01
TRBV19	ap01	01, 02, 03
TRBV2	ap01	01, 02, 03
TRBV20-1	ap01	01, 02, 03, 05, 06, 07
TRBV20-1	ap02	04
TRBV20/OR9-2	ap01	01, 02, 03
TRBV21-1	ap01	01, 02
TRBV21/OR9-2	ap01	01
TRBV23-1	ap01	01
TRBV23/OR9-2	ap01	01, 02
TRBV24-1	ap01	01, 02
TRBV25-1	ap01	01
TRBV26	ap01	01
TRBV26/OR9-2	ap01	01, 02

TRBV27	ap01	01
TRBV28	ap01	01
TRBV29-1	ap01	01, 02
TRBV29-1	ap02	03
TRBV29/OR9-2	ap01	01, 02
TRBV3-1/TRBV3-2	ap01	TRBV3-1*01, TRBV3-1*02, TRBV3-2*01, TRBV3-2*02, TRBV3-2*03
TRBV30	ap01	01, 03
TRBV30	ap02	02, 04
TRBV30	ap03	05
TRBV4-1	ap01	01, 02
TRBV4-2	ap01	01
TRBV4-2	ap02	02
TRBV4-3	ap01	01, 02, 03, 04
TRBV5-1	ap01	01, 02
TRBV5-3	ap01	01, 02
TRBV5-4	ap01	01, 02, 03, 04
TRBV5-5	ap01	01, 02, 03
TRBV5-6	ap01	01
TRBV5-7	ap01	01
TRBV5-8	ap01	01, 02
TRBV6-1	ap01	01
TRBV6-2/TRBV6-3	ap01	TRBV6-2*01, TRBV6-3*01
TRBV6-4	ap01	01, 02
TRBV6-5/TRBV6-6	ap01	TRBV6-5*01, TRBV6-6*01, TRBV6-6*02, TRBV6-6*03
TRBV6-5/TRBV6-6	ap02	TRBV6-6*04
TRBV6-5/TRBV6-6	ap03	TRBV6-6*05
TRBV6-7	ap01	01
TRBV6-8	ap01	01
TRBV6-9	ap01	01
TRBV7-1	ap01	01
TRBV7-2	ap01	01, 02, 04
TRBV7-2	ap02	03
TRBV7-3	ap01	01, 05

TRBV7-3	ap02	02
TRBV7-3	ap03	03
TRBV7-3	ap04	04
TRBV7-4	ap01	01
TRBV7-6	ap01	01, 02
TRBV7-7	ap01	01, 02
TRBV7-8	ap01	01
TRBV7-8	ap02	02
TRBV7-8	ap03	03
TRBV7-9	ap01	01, 02, 03, 04
TRBV7-9	ap02	05
TRBV7-9	ap03	06
TRBV7-9	ap04	07
TRBV9	ap01	01, 02, 03

Table S4: The distribution parameters of the TRBD2*01 fraction according to the TRBD2 genotype group in DS4.

TRBD2 genotype group \ TRBD2*01 fraction	Average	Median	Standard deviation
TRBD2*01 homozygous	0.96	0.96	0.003
TRBD2 heterozygous	0.453	0.452	0.022
TRBD2*02 homozygous	0.127	0.127	0.008

Table S5: **Previously unknown alleles comparison.** The left column is the unknown alleles of DS1 and DS2, columns 2-5 indicate if the allele was found in another dataset, i.e., DS3, DS4, Lou et al. [9], and pmTRIG [5], respectively. Column 6 indicates if the undocumented allele was observed in the long-read assemblies. The colors red, green, and blue in the first column correspond to alleles not following the expected multi-modal distribution, alleles adjacent to nucleotide stretches, or both, respectively. The purple color in the first column corresponds to an allele found in more than one data-set. For DS3 and DS4, it is impossible to identify undocumented SNPs that are outside the amplified region. Hence, out of the 24 undocumented alleles, 7 and 18 were out of range for DS3 and DS4, respectively.

DS1 [4] and DS2 [1]	DS3 [10]	DS4 [2]	Luo et al. [9]	pmTRIG [5]	long-read assemblies
TRBV10-1*02_G274T	TRBV10-1*bp02_G274T		TRBV10-1*02_gt234E_		True
TRBV6-6*01_G264A					
TRBV7-6*01_G297A					
TRBV12-4*01_C87T				TRBV12-4_3	True
TRBV12-5*01_C28G			TRBV12-5*01_cg27HD		True
TRBV12-5*01_C28G_T140A					
TRBV13*01_A170T					
TRBV13*01_T158C					
TRBV15*02_G275A					
TRBV19*01_A24G			TRBV19*01_ag23PP	TRBV19_3	True
TRBV20-1*01_C142A				TRBV20-1_4	
TRBV30*01_A113C					
TRBV5-6*01_T284G	TRBV5-6*bp01_T284G		TRBV5-6*01_tg244LW	TRBV5-6_4	True
TRBV5-8*01_C56T_C276T	TRBV5-8*bp01_C276T		TRBV5-8*01_ct55AV_ct236NN		
TRBV6-2/TRBV6-3*01_G47A					
TRBV6-6*01_C261T	TRBV6-6*bp01_C261T				
TRBV6-9*01_A303G			TRBV6-9*01_ag263VV		
TRBV7-7*01_C315T	TRBV7-7*bp01_C315T	TRBV7-7*ap01_C315T		TRBV7-7_2	True
TRBV10-2*01_C214T				TRBV10-2_3	
TRBV29-1*01_A280C			TRBV29-1*01_ac246ML	TRBV29-1_2	True
TRBV10-3*01_C225G					
TRBV4-1*01_C14T				TRBV4-1_6	
TRBV4-3*01_C274T					
TRBV7-3*01_G301A		TRBV7-3*ap01_G301A		TRBV7-3_5	True

Table S6: **Incomplete allele extensions table.** The left column is the call of the undocumented allele candidate, the second column is the matched incomplete allele reference in IMGT [7], and the third column is the length difference between the sequence of the undocumented allele candidate and the incomplete allele reference

Undocumented allele candidate	Incomplete allele	Length difference
TRBV10-3*01_T114C_G156A	TRBV10-3*03	14
TRBV14*01_G204A	TRBV14*02	5
TRBV2*01_G65A	TRBV2*02	5
TRBV20-1*05_A142C	TRBV20-1*02	5
TRBV5-5*01_A54C	TRBV5-5*02	4

Table S7: **5′ UTR variants.** The left column is the allele for which the 5′ UTR variant was found. The second column indicates if more than one consensus sequence was found for the allele. The third column is the number of samples in the cluster. The fourth and fifth columns indicate the 5′UTR SNP position and change for L-PART2 and L-PART1. The sixth column indicates if the variation is from alternative splicing events and shows the alternative sequence.

Allele	Consensus allele	cluster count	L-PART2 variation	L-PART1 variation	Alternative splicing variation
TRBV12-4*01	TRBV12-4*01_2	22		G5A	
TRBV13*01	TRBV13*01_2	9	T1G		
TRBV13*01	TRBV13*01_3	25	T1G	A53G	
TRBV18*01	TRBV18*01_2	15		G13C	
TRBV19*01	TRBV19*01_2	14		T37C	
TRBV19*01_A24G		6		T37C	
TRBV23-1*01	TRBV23-1*01_1	17			GACATTCTCTTTCTTTGTCTACGACATCTTTC TCAGGTCCTTTCTCGAATTGTTTGTTTTGTTT TGTTTTGTTTTGTTTTGTTTTGTTTCGAACCT AGACGACCAACCGTTCGGGTCCTGTTCCT AAAAAGAAAACACGAGTCCCGCGTA
TRBV23-1*01	TRBV23-1*01_2	6			GACATTCTCTTTCTTTGTCTACGACATCTTT CTCAGGTCCTTTCTCGAATTGTTTGTTTTGT TTTGTTTTGTTTTGTTTTGTTTTGTTTTGTT TCGAACCTAGACGACCAACCGTTCGGGTCCT GTTCCTAAAAAGAAAACACGAGTCCCGCGTA
TRBV25-1*01	TRBV25-1*01_2	15		A25G	
TRBV28*01		28		G18C	
TRBV5-4*01	TRBV5-4*01_2	8		T26C	
TRBV5-8*01_C56T_C276T	TRBV5-8*01_C56T_C276T_2	2		C47T	
TRBV6-2/TRBV6-3*01	TRBV6-2/TRBV6-3*01_2	10		G26A,G27C	
TRBV6-2/TRBV6-3*01	TRBV6-2/TRBV6-3*01_3	8		C29T	
TRBV6-2/TRBV6-3*01_G47A		1		G26A,G27C	
TRBV6-9*01		2		A47C,G48A	
TRBV6-9*01_A303G		2		A47C,G48A	
TRBV7-3*02		4		G12C	
TRBV7-7*01		18			ATTCTGTTTCCACAG
TRBV7-7*01_C315T	TRBV7-7*01_C315T_1	16			ATTCTGTTTCCACAG

Table S8: **5′ UTR undocumented sequences.** The left column is the allele for which the 5′ UTR variant was undocumented. The second column indicates which L-PART sequence is absent from IMGT. The third column is the number of samples in the cluster. The fourth column indicates which L-PART is also observed in the long read genomic assembly data-set.

Allele	Absent sequences	cluster count	Observed sequences in long-read assemblies
TRBV10-2*02	L-PART1 and L-PART2	7	L-PART1 and L-PART2
TRBV10-3*02	L-PART1	19	
TRBV10-3*03	L-PART1 and L-PART2	11	L-PART2
TRBV11-3*04	L-PART1 and L-PART2	11	L-PART1 and L-PART2
TRBV14*02	L-PART1 and L-PART2	8	L-PART2
TRBV15*02	L-PART1 and L-PART2	1	L-PART2
TRBV15*02_G275A	L-PART1 and L-PART2	1	
TRBV2*02	L-PART1 and L-PART2	1	L-PART1 and L-PART2
TRBV20-1*05	L-PART1 and L-PART2	6	L-PART1
TRBV3-2*03	L-PART1 and L-PART2	2	
TRBV5-5*02	L-PART1 and L-PART2	23	

Table S9: **Undocumented allele verification in artificial partial libraries.** To assess the ability of the presented pipeline to infer partial TRBV undocumented alleles from partial VDJ sequences, we generated from DS1 two artificial data-sets that simulate TRB repertoires obtained by BIOMED-2 and Adaptive Biotechnologies. For generating DS1 to stimulate a data-set obtained by BIOMED-2 protocol sequencing, the input sequences after the first alignment (see methods section 3.4) were trimmed by using the TRBV germline positions that the BIOMED-2 primers bind to. Sequences that aligned to TRBV genes whose BIOMED-2 primers do not bind were filtered out. Generating DS1 as a data-set obtained by Adaptive Biotechnologies pipeline was done by taking 87 nt up to the 23rd germline position from the end of the TRBJ gene whose sequence was assigned to. Next, we ran the trimmed sequences of both simulated data-sets through the pipeline. The table show the successful identification of the full length alleles by the artificial partial libraries. The left column is the undocumented allele from the complete DS1, the second and third columns are the artificial DS1 undocumented alleles for BIOMED2 and Adaptive lengths, respectively. The † indicated an undocumented allele inference which is identical to a known allele within the reference and hence did not make it to the genotype. The purple color indicates an allele that carries a SNP that is also found within the full length sequence. However, due to noise in the beginning of the full length sequences, there is no exact match to the undocumented allele version. This prevents the undocumented allele from passing the filtration step and enter the genotype, the same as in the artificial BIOMED2 dataset. This issue is the outcome of the initial sequence length and will persist in non-artificial sequences.

DS1 [4] undocumented alleles	DS1 [4] as BIOMED2	DS1 [4] as Adaptive
TRBV12-5*01_C28G	not applicable	not applicable
TRBV5-8*01_C56T_C276T	TRBV5-8*bp01_C276T	not applicable
TRBV6-9*01_A303G	TRBV6-9*bp01_A303G	TRBV6-9*ap01_A303G†
TRBV7-7*01_C315T	TRBV7-7*bp01_C315T	TRBV7-7*ap01_C315T
TRBV12-4*01_C87T	not applicable	not applicable
TRBV19*01_A24G	not applicable	not applicable
TRBV6-2/TRBV6-3*01_G47A	not applicable	not applicable
TRBV15*02_G275A	TRBV15*bp02_G275A	not applicable
TRBV12-5*01_C28G_T140A	not applicable	not applicable
TRBV5-6*01_T284G	TRBV5-6*bp01_T284G	not applicable
TRBV10-1*02_G274T	TRBV10-1*bp02_G274T	not applicable
TRBV6-6*01_G264A	TRBV6-6*bp01_G264A	not applicable
	TRBV10-2*bp01_T233C	
	TRBV6-9*bp01_A229C_A303G	

Table S10: **Previously unknown alleles comparison.** The left column is the unknown alleles of DS3, columns 2-5 indicate if the allele was found in another dataset, DS1 and DS2, DS4, Lou et al. [9], and pmTRIG [5], respectively. Column 6 indicates if the undocumented allele was observed in long-read assemblies. The colors red, green, and blue in the first column correspond to alleles not following the expected multi-modal distribution, alleles adjacent nucleotide stretches, or both, respectively. The purple color in the first column corresponds to an allele found in more than one dataset.

DS3 [10]	DS1 [4] and DS2 [1]	DS4 [2]	Luo et al. [9]	pmTRIG [5]
TRBV10-1*bp01_C190A_C195T_A199G				
TRBV10-1*bp01_G274T				
TRBV10-1*bp02_G156A_G274T				
TRBV10-1*bp02_G274T	TRBV10-1*02_G274T		TRBV10-1*02_gt234	
TRBV11-1*bp01_C149T				TRBV11-1_5
TRBV11-3*bp01_G297A				
TRBV15*bp02_G153T				
TRBV19*bp01_T310C_G311C_C314T				
TRBV24-1*bp01_A316C				
TRBV24-1*bp01_G252A				
TRBV29-1*bp03_C315T				
TRBV30*bp01_C253T				
TRBV30*bp01_G298A		TRBV30*ap01_G298A		TRBV30_8
TRBV30*bp02_C237T				
TRBV5-4*bp01_G205A				
TRBV5-4*bp01_G205A				
TRBV5-5*bp01_T284G_G303C				
TRBV5-6*bp01_T284G	TRBV5-6*01_T284G		TRBV5-6*01_tg244	TRBV5-6_4
TRBV5-8*bp01_C276T	TRBV5-8*01_C56T_C276T		TRBV5-8*01_ct236	
TRBV6-4*bp01_C269G				
TRBV6-5*bp01_G279A				
TRBV6-6*bp01_C261T	TRBV6-6*01_C261T			TRBV6-6_8
TRBV6-6*bp01_G256T				TRBV6-6_4
TRBV6-9*bp01_G155T_C156G_A303G				
TRBV7-4*bp01_G251A			TRBV7-4*01_ga214	TRBV7-4_3
TRBV7-4*bp01_T306C_C307T				
TRBV7-7*bp01_C315T	TRBV7-7*01_C315T	TRBV7-7*ap01_C315T		TRBV7-7_2
TRBV7-7*bp01_T273C				
TRBV7-9*bp04_T312A				

Table S11: **Previously unknown alleles comparison.** The left column is the unknown alleles of datasets DS4, columns 2-5 indicates if the allele was found in another dataset, DS1 and DS2, DS3, Lou et al. [9], and pmTRIG [5], respectively. Column 6 indicates if the unknown allele was observed in long-read assemblies. The colors red, green, and blue in the first column correspond to alleles not following the expected multi-modal distribution, alleles adjacent nucleotide stretches, or both, respectively. The purple color in the first column corresponds to an allele found in more than one dataset.

DS4 [2]	DS1 [4] and DS2 [1]	DS3 [10]	Luo et al. [9]	pmTRIG [5]	long-read assemblies
TRBV11-3*ap01_T312C					
TRBV15*ap02_C296T					
TRBV15*ap02_G290C					
TRBV20-1*ap01_G314A					
TRBV20-1*ap02_T310G					
TRBV25-1*ap01_A293G				TRBV25-1_5	
TRBV28*ap01_A297G					
TRBV30*ap01_G298A		TRBV30*bp01_G298A		TRBV30_8	
TRBV4-3*ap01_A305C_T306C					
TRBV4-3*ap01_A305C_T306C_T308C					
TRBV4-3*ap01_G311C_G313C					
TRBV4-3*ap01_T308C_G311C					
TRBV4-3*ap01_T308C_T310C_G311C					
TRBV6-8*ap01_A293G			TRBV6-8*01_ag250		
TRBV7-3*ap01_G292T			TRBV7-3*01_gt255	TRBV7-3_4	True
TRBV7-3*ap01_G301A	TRBV7-3*01_G301A			TRBV7-3_5	
TRBV7-4*ap01_G291C_A297G					
TRBV7-4*ap01_G291C_A297G_C314T					
TRBV7-6*ap01_C314G					
TRBV7-7*ap01_C307A_C315T					
TRBV7-7*ap01_C307T_C315T					
TRBV7-7*ap01_C315T	TRBV7-7*01_C315T			TRBV7-7_2	
TRBV7-8*ap01_T295C			TRBV7-8*01_tc258		
TRBV7-9*ap01_G313T					

References

[1] 10x Genomics. 10x datasets. `https://support.10xgenomics.com/single-cell-vdj/datasets`. Accessed: 2020-12-15.

[2] Adaptive Biotechnologies. Adaptive biotechnologies datasets. `https://clients.adaptivebiotech.com/pub/Emerson-2017-NatGen`. Accessed: 2020-12-15.

[3] Ron Edgar, Michael Domrachev, and Alex E Lash. Gene expression omnibus: Ncbi gene expression and hybridization array data repository. *Nucleic acids research*, 30(1):207–210, 2002.

[4] Sivan Eliyahu, Oz Sharabi, Shiri Elmedvi, Reut Timor, Ateret Davidovich, Francois Vigneault, Chris Clouser, Ronen Hope, Assy Nimer, Marius Braun, Yaacov Y. Weiss, Pazit Polak, Gur Yaari, and Meital Gal-Tanamy. Antibody repertoire analysis of hepatitis c virus infections identifies immune signatures associated with spontaneous clearance. *Frontiers in Immunology*, 9:3004, 2018.

[5] Indu Khatri, Magdalena A. Berkowska, Erik B. van den Akker, Cristina Teodosio, Marcel J.T. Reinders, and Jacques J.M. van Dongen. Population matched (pm) germline allelic variants of immunoglobulin (ig) loci: New pmig database to better understand ig repertoire and selection processes in disease and vaccination. *bioRxiv*, 2020.

[6] Ilkka Lappalainen, Jeff Almeida-King, Vasudev Kumanduri, Alexander Senf, John Dylan Spalding, Gary Saunders, Jag Kandasamy, Mario Caccamo, Rasko Leinonen, Brendan Vaughan, et al. The european genome-phenome archive of human data consented for biomedical research. *Nature genetics*, 47(7):692–695, 2015.

[7] Marie-Paule Lefranc, Véronique Giudicelli, Chantal Ginestoux, Julia Bodmer, Werner Müller, Ronald Bontrop, Marc Lemaitre, Ansar Malik, Valérie Barbié, and Denys Chaume. Imgt, the international immunogenetics database. *Nucleic Acids Research*, 27(1):209–212, 1999.

[8] Mingfeng Liao, Yang Liu, Jing Yuan, Yanling Wen, Gang Xu, Juanjuan Zhao, Lin Cheng, Jinxiu Li, Xin Wang, Fuxiang Wang, Lei Liu, Ido

Amit, Shuye Zhang, and Zheng Zhang. Single-cell landscape of bronchoalveolar immune cells in patients with covid-19. *Nature Medicine*, 26(6):842–844, Jun 2020.

[9] Shishi Luo, Jane A Yu, Heng Li, and Yun S Song. Worldwide genetic variation of the ighv and trbv immune receptor gene families in humans. *Life Science Alliance*, 2(2), 2019.

[10] Donjete Simnica, Nuray Akyüz, Simon Schliffke, Malte Mohme, Lisa v.Wenserski, Thorben Mährle, Lorenzo F. Fanchi, Katrin Lamszus, and Mascha Binder. T cell receptor next-generation sequencing reveals cancer-associated repertoire metrics and reconstitution after chemotherapy in patients with hematological and solid tumors. *OncoImmunology*, 8(11):e1644110, 2019. PMID: 31646093.

[11] Wen Wen, Wenru Su, Hao Tang, Wenqing Le, Xiaopeng Zhang, Yingfeng Zheng, Xiuxing Liu, Lihui Xie, Jianmin Li, Jinguo Ye, Liwei Dong, Xiuliang Cui, Yushan Miao, Depeng Wang, Jiantao Dong, Chuanle Xiao, Wei Chen, and Hongyang Wang. Immune cell profiling of covid-19 patients in the recovery stageby single-cell sequencing. *Cell Discovery*, 6(1):31, May 2020.
